# Daily Beneficial Effects of Work-to-Family Facilitation on Employees' Recovery and General Health: Is More Work Engagement Always Better?

**DOI:** 10.3389/fpsyg.2021.661267

**Published:** 2021-07-15

**Authors:** Isabel Carmona-Cobo, Luis Manuel Blanco-Donoso, Eva Garrosa

**Affiliations:** ^1^Department of Psychology, University of Jaén, Jaén, Spain; ^2^Department of Biological and Health Psychology, Autonomous University of Madrid, Madrid, Spain

**Keywords:** daily diary study, detachment from work, health, recovery, relaxation, work engagement, work-to-family facilitation, work-home resources model

## Abstract

This study of 104 Chilean employees examines the process of WFF—recovery—general health on a daily basis. Drawing on the work–home resources (W-HR) model, we hypothesized that daily work-to-family facilitation and work engagement predict recovery experiences during off-job time in the evening (i.e., detachment from work and relaxation) and subsequent general health at night. Furthermore, we explored whether daily work engagement moderates the relationships between daily work-to-family facilitation and recovery experiences during off-job time in the evening and general health at night. In addition, we expected employees' detachment from work to have a lagged effect on next-day general health at night. Participants completed a survey and a diary booklet over 5 consecutive working days (*N* = 520 occasions). Multilevel analyses show that, as expected, daily work-to-family facilitation predicted recovery experiences during off-job time in the evening (i.e., detachment from work and relaxation). However, contrary to our expectations, daily work engagement only predicted general health at night. Moreover, as expected, a moderation effect of daily work engagement shows that on days that employees experience low levels of daily work engagement, daily work-to-family facilitation is strongly related to detachment from work and relaxation during off-job time in the evening and to general health at night. Unexpectedly, on days on which employees experienced high levels of daily work engagement, daily work-to-family facilitation was weakly related to these outcomes. Finally, in accordance with our expectations, detachment from work had a lagged effect on next-day general health at night. These findings offer support for the W-HR model and have theoretical and practical implications for research and organizations.

## Introduction

Changing and forthcoming work demands a focus on the need to facilitate work-to-home functioning that fosters constructive and balanced relationships between these spheres to facilitate employees' recovery from work and health. Usually, research on the positive side of the work–family interface has focused on identifying several related but separate constructs, such as work-family facilitation (WFF), positive spillover, and enrichment. WFF refers to the enhancement system functioning processes by which individual participation in one role (e.g., home) is improved by the skills or competencies acquired in the other role (e.g., work) (Frone, 2003). Wayne and colleagues defined WFF as “the extent to which an individual's engagement in one life domain (i.e., work/family) provides gains (i.e., developmental, affective, capital, or efficiency) which contribute to enhanced functioning of another life domain (i.e., family/work)” (Wayne et al., 2007, p. 64).

Due to enhanced system functioning, the gains obtained from a work system can enhance the functioning of the family system. Four broad categories of gains have been conceptualized: (1) developmental gains, referring to skills, knowledge, values, or perspectives; (2) affective gains, referring to aspects of emotion; (3) capital gains, referring to economic, social, or health assets; and (4) efficiency gains, referring to the enhanced focus or attention induced by multiple role responsibilities. The theoretical framework of this study is situated in the context of conservation of resources (COR) theory (Hobfoll, 2001). This theory posits that employees have limited resources; hence, if employees spend such resources in the work sphere, there are only limited resources left to be dedicated to the home sphere. Building on this idea and expanding on knowledge of the work-to-family positive direction, the present study stems from COR theory and uses the work–home resources (W-HR) model (ten Brummelhuis and Bakker, 2012) to explain the daily beneficial effect of daily WFF as a short-term process of gain spirals.

Regarding other similar but separate constructs, positive spillover refers to the process by which what happens in one domain often spills back over to the other domain. These processes imply a transference of the acquisition of gains in one domain (e.g., work) to the use of these gains in the other domain (e.g., home; Grzywacz and Marks, 2000). Finally, from the theoretical model of work-family enrichment developed by Greenhaus and Powell (2006), the construct of enrichment refers to “the extent to which experiences in one role improves the quality of life in the other role” (Greenhaus and Powell, 2006, p. 73). Hence, the processes of enrichment imply enhanced individual functioning. Research has sometimes used these related terms interchangeably because of their conceptual ambiguity and confusion within the field on the positive synergistic potential of work and family experiences (Wayne, 2009). However, as one major distinction between these concepts, WFF has beneficial effects at system-level functioning, whereas the individual specificity of positive spillover benefits from the transference of gains, and the enrichment benefits improve individual-level functioning.

Despite the larger body of research on WFF in different cultures (Jeffrey Hill et al., 2004), there is a gap in the knowledge about its beneficial effects in the Chilean context. Traditionally, the literature on work-family balance in Chile has demonstrated the family orientation of this society, also showing that women mainly occupy the family sphere, as they are responsible for family-related tasks (Staab, 2012). However, recent social changes in demographic and family trends offer a new configuration in which participation in family-related tasks constitutes coresponsibility demands (Jiménez Figueroa and Gómez Urrutia, 2014). Recent insights suggest that the positive processes of WFF are also a relevant theme to be explored in this context. For example, a study conducted by Jiménez Figueroa et al. (2017) on Chilean workers showed that participation in family responsibilities is positively related to work-family balance and that positive balance is associated with parental self-efficacy. Moreover, Orellana et al. (2021) explored Chilean dyads of different-sex dual-earner parents and found that family support is positively associated with life satisfaction *via* work-life balance. On the basis of nascent research on WFF in the Chilean context, this study fits within the extant literature to better explore its benefits in a daily short period of time.

Based on W-HR theory, this research explores the daily process of WFF—recovery—general health among Chilean employees. The aim was to analyze the daily beneficial effects of day-level WFF and daily work engagement on recovery experiences during off-job time in the evening (i.e., detachment from work and relaxation) and general health at night in a work week as well as the lagged effect of detachment from work on next-day general health at night. The present study contributes to the existing literature in several ways. First, given the scarce day-level analyses of previous research on the positive daily beneficial effect of WFF on recovery experiences and general health, the present study fills this gap by conducting a one-week diary study to capture changes occurring within relatively short time intervals from work to home (Ohly et al., 2010). Second, although research focuses on the positive side of daily work engagement, mostly confirming benefits in job performance, the present study adds findings to the scarce evidence on its daily effects as a predictor and moderator of recovery experiences during off-job activities and subsequent general health at night to determine the level of daily work engagement needed to support an individual's well-being (Shimazu et al., 2018). Third, as detachment from work is the core recovery experience associated with several benefits for employees (e.g., emotional well-being; Garrosa-Hernández et al., 2013), our study tests the unknown lagged effect of detachment from work on next-day general health at night instead of the well-known moderation and mediational roles.

### Daily Beneficial Effect of WFF on Recovery and General Health

When considering recovery as the process of restoring employees' resources (Zijlstra and Sonnentag, 2006; Sonnentag and Fritz, 2007), the importance of work-to-family balance for the recovery process seems crucial. Until now, most findings have focused on the positive outcomes of daily recovery experiences for employees' well-being instead of their antecedents (Blanco-Donoso et al., 2017; Wendsche and Lohmann-Haislah, 2017; Bennett et al., 2018). Additionally, experimental studies have been conducted to test the affective consequences of daily recovery experiences (Sonnentag and Niessen, 2020). Currently, new insights suggest the pertinence of exploring daily fluctuations in the antecedents of recovery experiences due to emerging trends in the work sphere (Rodríguez-Muñoz et al., 2018; Chawla et al., 2020; Pfaffinger et al., 2020). A meta-analysis of antecedents and outcomes of detachment from work found that this recovery experience is influenced by work-related and personal characteristics (Wendsche and Lohmann-Haislah, 2017). Moreover, work events and physiological indicators of parasympathetic regulation have been identified as important antecedents of off-the-job relaxation (Parker et al., 2020).

**Figure 1:**
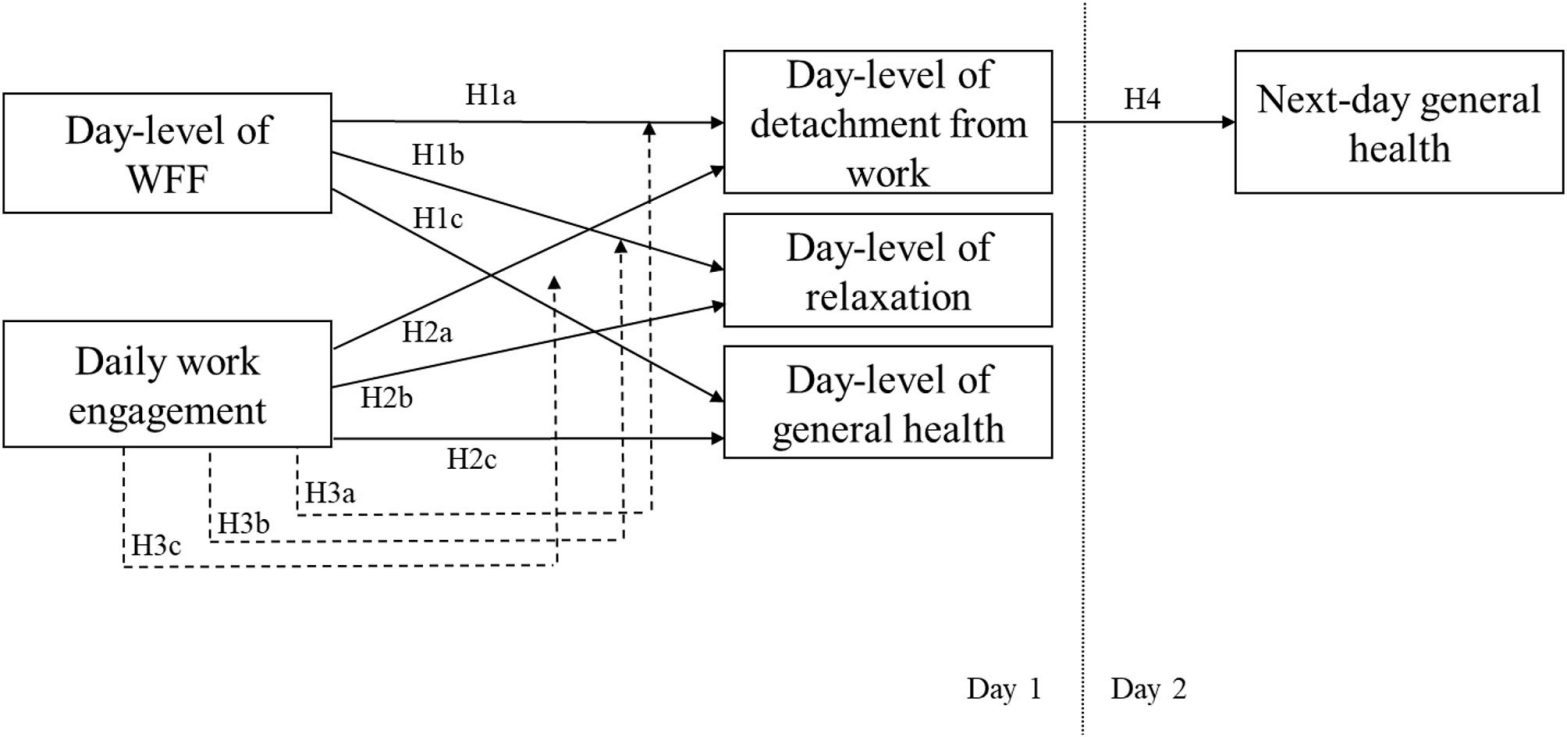
Research day-level model for testing hypotheses within the same and next-day.

The W-HR model (ten Brummelhuis and Bakker, 2012) may help explain how short-term work-home processes of day-level WFF can benefit recovery processes on the daily basis. Day-level WFF can be described as a process of resource accumulation by means of work resources that can increase personal resources and where those personal resources, in turn, can be utilized to improve home system functioning, such as through recovery experiences and being healthy. Thus, day-level WFF occurs when the developmental, affective, capital and efficiency gains acquired in the work system are transferred to and enhance the functioning of the family system, with day-level WFF being positively related to the day-to-day recovery process (Wayne et al., 2007). Based on the W-HR model (ten Brummelhuis and Bakker, 2012), the present study examines the beneficial effects of day-level WFF on recovery experiences, suggesting that this positive process may operate within a relatively short time frame on a daily basis, fostering subsequent general health at night. We examine whether employees experience a higher level of detachment from work and relaxation during off-job time in the evening and increase their level of general health at night on days involving higher levels of WFF. Thus, we propose the following (please, see Figure 1):

*Hypothesis 1:* Day-level WFF will be positively related to (H1a) detachment from work, (H1b) relaxation during off-job time in the evening, and (H1c) general health at night.

### Effect of Daily Work Engagement on Recovery and General Health

Work engagement is a well-documented predictor of important outcomes at the employee, team, and organizational levels (Bakker and Albrecht, 2018). Currently, it is assumed that work engagement fluctuates among employees over time (Sonnentag et al., 2010b). For example, daily fluctuations are found in workers who are engaged during workdays when they have reached a high level of recovery during evenings (Sonnentag, 2003). In fact, a reciprocal relationship has been demonstrated between daily work engagement and daily recovery (Bakker, 2014). This means that on days on which employees recover well, they are also more engaged, and on days on which employees feel engaged during the day, they experience subsequent daily recovery (Sonnentag et al., 2012). Daily work engagement refers to daily levels of vigor, dedication, and absorption that may fluctuate as a function of daily demands, resources, and proactive behaviors (Bakker and Albrecht, 2018). From this perspective, it can reasonably be expected that daily work engagement promotes recovery experiences and fosters general health as a consequence. On the basis of the W-HR model (ten Brummelhuis and Bakker, 2012), gain spirals of daily work engagement may explain how on days on which employees are engaged in the work sphere, they can use the rest of their energy during off-job time through recovery experiences, transferring engagement to participation in the home sphere. In this sense, we expect to find positive direct effects of daily work engagement on the level of recovery during leisure time and on general health at night. Thus, we propose the following:

*Hypothesis 2:* Daily work engagement will be positively related to (H2a) detachment from work, (H2b) relaxation during off-job time in the evening, and (H2c) general health at night.

Empirical research has reported the moderating role of work engagement in recovery, for example, in buffering the negative effect of workload on psychological detachment from work (Clauss et al., 2020). However, little is known about the adequate level of daily work engagement that moderates relationships, resulting in positive outcomes such as recovery and general health (Blanco-Donoso et al., 2021). An emerging line of research has introduced the debate on a “dark side of engagement” in the literature, which posits that more work engagement is not necessarily preferable (Bakker and Leiter, 2010; George, 2010; Bakker et al., 2011; Leiter, 2019). A study supporting this idea reveals adverse effects of high work engagement on psychological distress in the short term, showing a curvilinear relationship between work engagement and psychological distress (Shimazu et al., 2018). Hence, high levels of daily work engagement might have detrimental effects on individuals' well-being.

Drawing on the W-HR model (ten Brummelhuis and Bakker, 2012), short-term work engagement may reflect daily processes by means of employees who spend their volatile contextual resources in the work sphere (e.g., being highly engaged at work) and may affect daily outcomes in the home domain through a change in volatile personal resources (e.g., for recovering well during off-job time; Blanco-Donoso et al., 2021). To allow employees to use their gains from work to recover during off-job time in the evenings and even experience higher daily levels of general health at night, they might require a low level of daily work engagement during the workday instead of a high level, as it may drain their resources. Based on this idea, we examine the moderating role of daily work engagement and propose the following hypothesis:

*Hypothesis 3:* The relationship between day-level WFF and (H3a) detachment from work, (H3b) relaxation during off-job time in the evening, and (H3c) general health at night will be stronger when an employee's daily level of work engagement is lower (rather than higher).

### Lagged Effect of Detachment From Work on Next-Day General Health

Detachment from work has been considered a mediator of the relations between employees' responses to stressful work-related experiences and health (Sonnentag and Fritz, 2015). Beyond physical distance to the workplace, detachment from work refers to a withdrawal from work not only physically but also psychologically. Thus, psychological processes are key to detachment from work, implying not thinking about work or doing work-related activities outside of the workplace (Sonnentag and Fritz, 2007). The benefits of detachment from work go further than the individual emotional outcomes experienced on the same day at night (e.g., Garrosa et al., 2015) and affect the next day's level of well-being. In particular, several studies have shown the lagged effects of detachment from work in the following morning. For example, the level of work detachment in the evening predicts low levels of negative affect and fatigue (Sonnentag et al., 2008), high levels of vigor (ten Brummelhuis and Bakker, 2012), and feelings of recovery (Volman et al., 2013), and a lack of psychological detachment has been related to near accidents when commuting to work the next morning (Pereira et al., 2016).

Despite previous evidence, it is still unknown whether the lagged effect of detachment from work in the evening on the level of well-being persists at the end of the following day. Following the W-HR model (ten Brummelhuis and Bakker, 2012), the daily level of detachment from work during off-job time may reflect short-term processes of gaining spirals within the home sphere to maintain optimal general health. Day-level detachment may act as a daily outcome that operates on the next day. Thus, on days on which employees have more gains and reach high levels of detachment from work during off-job time, they are also more likely to experience better general health due to the daily process of restoring resources, with benefits persisting to the next day. Considering the daily process, the present study examines whether detachment from work during off-job time in the evening on day 1 predicts employees' general health the following day at night. Thus, we propose the following hypothesis:

*Hypothesis 4:* The daily level of detachment from work during off-job time in the evening will be positively related to next-day general health at night.

## Materials and Methods

### Sample and Procedure

We collected data from 104 Chilean employees from a variety of industries, including education (26%), administration (19.2%), services (21.2%), health (6.7%), banking (1.9%), and Information Technology and Communications (1.9%), among others (23.1%). Data were collected before the COVID-19 outbreak. The sample was composed of 43.3% men and 56.7% women with a mean age of 42.3 (*SD* = 16.5) of a range of ages including 20–30 (32%), 31–40 (20%), 41–50 (23%), and 51–60 (25%). With respect to family status, 47.1% of the participants were married or living with a partner, and 35.6% were single. Among the participants, 63.4% had children (16.3% had one child, 26% had two children, 16.3% had three children, and 4.8% had four children). Regarding education level, 37.1% held a university degree, and 36.5% held a technical degree. Most of the participants worked for small and medium enterprises (54.8%), and the mean job tenure was 18.0 years (*SD* = 26.7). Most of the participants had a permanent contract (63.5%) and a full-time schedule (78.8%) and worked 44 or 45 h per week (46.2%). A snowball sampling technique was used to recruit participants from several local organizations with the following inclusion criterion: working with a timetable of 5 consecutive days during the work week. We based this criterion on methodology recommendations made by Ohly et al. (2010) for diary studies in organizational research. Following similar diary studies evaluating employees, we selected serial temporary data for 5 working days to capture an entire weekly work cycle (i.e., Sanz-Vergel et al., 2010a; Garrosa et al., 2015). After the participants expressed their willingness to voluntarily participate in the study, the researchers gave them and explained a paper-based package that included (a) a letter describing the objective of the study, instructions on the completion of the surveys, and simple questions used to create a personal code to preserve anonymity (i.e., the year of birth); (b) two informed consent forms, with one copy for researchers and another copy for the participants; and (c) the general and daily paper-based questionnaires. The participants did not receive a reward for taking part in the study. Ethical permission was obtained and approved by the Research and Ethics Committee at Temuco Catholic University (Chile). Participants were instructed to complete the general questionnaire (Level 2, Person-level) before starting on the daily questionnaires (Level 1, Day-level) for 5 consecutive working days twice a day (*N* = 520 observations).

To capture daily dynamic processes (Bolger et al., 2003), the researchers designed diary entry intervals to better understand work and non-work periods of the participants' schedules. Specifically, the participants completed Monday through Friday daily work engagement in the afternoon, immediately after returning home from work and referring to the workday; day-level WFF at night refers to home activities performed in the evening; day-level detachment from work and relaxation at night refers to off-job time in the evening; and general health at night was measured before bedtime. To ensure that the participants appropriately completed the questionnaires, the researchers underlined the importance of following the procedure, and the participants were also given a reminder to make daily entries. The time taken to complete the daily surveys was also recorded. The participants completed the afternoon form at 18:18 p.m. on average (*SD* = 43 min) and the nighttime form at 23:04 p.m. on average (*SD* = 1 h and 11 min).

### Measures

We used a general questionnaire to collect data at the individual level (Level 2, general) and daily questionnaires at the day level (Level 1, daily). As a previous step before administering the general and daily questionnaires, a group of academicians selected the Chilean versions of the scales and adapted the language of those for which there were no versions. Based on guidelines provided by Hambleton and De Jong (2003), all of the scales were revised by academicians focused on the Chilean context and on psychology and were further analyzed through group discussions to test their adequacy for studying Chilean employees (Willgerodt, 2003; Vogt et al., 2004). Then, experts reviewed the appropriateness of the items' use over the established timeline according to the sample characteristics. At the day level (Level 1), daily measures of predictor and criterion variables were modified from the corresponding general scale to the specific diary entries made at night (Ohly et al., 2010; Nezlek, 2012). Following previous studies, responses measured on 5-point Likert scales of 1 = *I fully disagree* to 5 = *I fully agree* were given for all items (Rodríguez-Muñoz et al., 2012).

#### Day-Level of WFF (Level 1)

We assessed positive transference from work to family by using subscale “Positive Interaction of Work-Family” from Work-Home Interaction Survey-Nijmegen (Geurts et al., 2005) translated into Spanish by Moreno-Jiménez et al. (2009). Participants responded at night by indicating which of the five items they had experienced at home in the evening due to work-related factors. Sample items were adapted to the Chilean context and referred to specific diary entries (for example, “Today was a pleasant workday, and I felt more in the mood to do activities with my partner/family/friends during my leisure time”). Strong average reliability was obtained over 5 days (α = 0.91, with a range of 0.90–0.93). The reliability and validity of the scale are well-established in terms of internal consistency and construct validity (Moreno-Jiménez et al., 2009).

#### Daily Work Engagement (Level 1)

This variable was measured with the Chilean version (Müller Gilchrist et al., 2013) of the Utrecht Work Engagement Scale (UWES; Schaufeli et al., 2006) adapted for day-level assessment in the afternoon immediately after returning home from work and referring to work days. The UWES evaluates three components of work engagement: vigor, dedication, and absorption. The scale includes 17 items referring to the present moment, such as “Today, at work, I felt strong and vigorous” (vigor); “Today, I was proud of the work that I did” (dedication); and “Today, time flied at work” (absorption). Strong average reliability was obtained over 5 days (α = 0.94, with a range of 0.93–0.95). The reliability and validity of the scale are well-established in terms of internal and construct validity (Müller Gilchrist et al., 2013).

#### Day-Level of Detachment From Work (Level 1)

We assessed the participants' nighttime psychological detachment levels with the four items of the subscale of the Spanish version of the Recovery Experiences Questionnaire (Sanz-Vergel et al., 2010b) from the original measure developed by Sonnentag and Fritz (2007). Participants responded at night on the degree to which they had experienced detachment during off-job time in the evening. An example psychological detachment work item, adapted to the Chilean context and with reference to the present moment at night, is “Today, when I left work, I didn't think about work at all.” Strong average reliability was obtained over 5 days (α = 0.91, with a range of 0.90–0.93). The reliability and validity of the scale are well-established in terms of internal consistency and construct validity (Sanz-Vergel et al., 2010b).

#### Day-Level of Relaxation (Level 1)

We used the Spanish version of the Recovery Experiences Questionnaire (Sanz-Vergel et al., 2010b) developed by Sonnentag and Fritz (2007). To assess at night the degree to which the participants had experienced relaxation during off-job time in the evening, we used the four relaxation subscale items. We adjusted all items for day-level measurement as well (for example, “Today, during my leisure time, I kicked back and relaxed”). Stronger average reliability was obtained over 5 days (α = 0.89, with a range of 0.87–0.91). The reliability and validity of the scale are well-established in terms of internal consistency and construct validity (Sanz-Vergel et al., 2010b).

#### Day-Level of General Health (Level 1)

We used the Chilean version of the GHQ-12 (Araya et al., 1992) to measure the daily level of employees' general health at night before going to bed. The GHQ-12 scores were calculated by averaging them. We adjusted the items for day-level measurement at night. A sample item is “Today, I was able to concentrate.” Sufficient average reliability was obtained over 5 days (α = 0.57, with a range of 0.50–0.63). The reliability and validity of the scale are well-established in terms of internal consistency and construct validity (Araya et al., 1992).

#### Daily (Level 1) and General (Level 2) Control Variables

At the individual level (Level 2, general), we assessed a person's general level of detachment from work, relaxation, and general health as features of criterion variables (Garrosa et al., 2017). At the day level (Level 1), we used daytime as a control variable for the work day of the 5 consecutive day-level measures (Ouweneel et al., 2012).

### Analytic Strategy

We used hierarchically structured data with repeated measures at the day level nested among individuals at the individual level simultaneously (Hox, 2010). The model has two levels: repeated measures at the day level (Level 1: within-person variation; *N* = 520 diary entries) and participants at general Level 2 (between-person variation; *N* = 104). To analyze the two-level dataset, we conducted a multilevel analysis using a hierarchical linear modeling approach (Nezlek, 2012). Based on Ohly et al. (2010), we centered the predictor daily variables of Level 1 (day-level WFF and daily work engagement) using the person-mean centering method, which considers the mean scores of each participant. The grand-mean centering method, which considers the mean scores of all participants, was used for the general or trait criterion variables and for predictor variables of Level 2. This procedure eliminates all of the variance between subjects so that it does not influence the interpretation of the results. Data were analyzed using MLwiN software (Leckie and Charlton, 2013).

## Results

### Descriptive and Preliminary Analyses

Before testing our hypotheses, we examined within- and between-person variation in our variables. When calculating the intraclass coefficient for the outcome variables, we found that within-person variance reached 66.5% for detachment from work, 64.5% for relaxation, and 71.1% for general health. The intraclass coefficient of the predictor variables was measured at 67.8% for day-level WFF and at 77.7% for daily work engagement. All predictor and criterion variables showed an intraclass correlation coefficient of above 25% (Hox and Roberts, 2011). Overall, these findings suggest that a substantial portion of the variance in our outcome variables can be attributed to within-person variation across the 5 days, which supports the use of multilevel analysis (Fisher and To, 2012). Table 1 shows the means, standard deviations, reliability of day-level and general measures, and bivariate correlations among the study variables.

**Table 1:** Means, standard deviation, Cronbach's alpha, and correlations between the person and day-level variables (*N* = 520 observations, *N* = 104 participants).

**Variables**	***M***	***SD***	**α**	***Skewness***	***Kurtosis***	**1**	**2**	**3**	**4**	**5**	**6**	**7**	**8**	**9**	**10**
1. General WFF	3.08	0.97	0.83	0.08	−0.72	-									
2. General work engagement	4.03	0.61	0.91	−0.82	0.80	14^**^									
3. General detachment from work	3.37	0.99	0.90	−0.39	−0.54	0.24^**^	−0.21^**^								
4. General relaxation	3.57	0.87	0.78	−0.21	−0.43	0.31^**^	0.07	0.55^**^							
5. General health	3.84	0.59	0.86	−0.29	−0.94	0.11^*^	0.41^**^	0.23^**^	0.30^**^						
6. Day-level of WFF	2.96	1.01	0.91	0.18	−0.44	0.45^**^	0.21^**^	0.13^**^	0.17^**^	−0.01					
7. Daily work engagement	3.61	0.73	0.94	−0.15	−0.37	0.19^**^	0.56^**^	−0.08	0.18^**^	0.29^**^	0.44^**^				
8. Day-level of detachment from work	3.40	1.02	0.91	−0.28	−0.47	0.13^**^	−0.07	0.55^**^	0.42^**^	0.23^**^	0.20^**^	0.17^**^			
9. Day-level of relaxation	3.42	1.02	0.89	−0.27	−0.50	0.28^**^	0.05	0.38^**^	0.47^**^	0.21^**^	0.34^**^	0.25^**^	0.67^**^		
10. Day-level of general health	3.87	0.66	0.57	−0.27	−0.37	0.04	0.39^**^	0.21^**^	0.24^**^	0.67^**^	0.07	0.46^**^	0.35^**^	0.30^**^	-

*WFF, Work-Family Facilitation*.

*The average reliability of Cronbach's alpha over 5 days is displayed for day-level variables*.

* *p <0.05*,

** *p <0.01*.

### Hypothesis Test

To test our hypotheses, we compared four nested models that included the specific general or trait (Level 2 person-level: between-person variation) and daily (Level 1 day-level: within-person variation) variables. The null model included only the intercept. In Model 1, we entered control variables at the individual level for Level 2 (e.g., general or trait criterion variables and day). In Model 2, we added daily WFF (Level 1) for testing H1. In Model 3, we included daily work engagement (Level 1) to test H2. In Model 4, we included the interaction term of the daily level of WFF (Level 1) as a predictor variable, and daily work engagement (Level 1) as a moderator for testing the moderation hypothesis (H3). In addition, we applied two nested hierarchical linear models to test the lagged effect hypothesis (H4). In this case, we included the null model with the intercept. In Model 1, we included employees' general health or health traits and day person-level variables (Level 2), and in Model 3, we added detachment from work as a predictor day-level variable (Level 1). To assess the improvement of each model over the previous one, we explored the differences between the respective likelihood ratios. Tables 2–**5** present model fit information (difference of −2 x Log) estimates for the fixed parameters and estimates for the variance components. As a measure of effect size, we computed pseudo-*R*^2^ following the recommendations of Singer and Willett (2003). The pseudo-*R*^2^ statistic is used to quantify the incremental variance in the dependent variable predicted by adding a new set of predictors to a given model.

**Table 2:** Multilevel estimates for models predicting day-level of detachment from work (*N* = 520 observations, *N* = 104 participants).

**Variables**	**Null Model**			**Model 1**			**Model 2**			**Model 3**			**Model 4**		
	**Estimate**	**SE**	***t***	**Estimate**	**SE**	***t***	**Estimate**	**SE**	***t***	**Estimate**	**SE**	***t***	**Estimate**	**SE**	***T***
Intercept	3.40	0.09	39.96^***^	3.40	0.07	52.26^***^	3.40	0.07	52.26^***^	3.40	0.07	52.26^***^	3.41	0.07	52.45^***^
General detachment from work				0.56	0.07	8.52^***^	0.56	0.07	8.52^***^	0.56	0.07	8.52^***^	0.56	0.07	8.42^***^
Day				0.01	0.02	0.56	0.01	0.02	0.72	0.02	0.02	0.89	0.02	0.02	0.94
Day-level of WFF							0.13	0.05	2.61^**^	0.11	0.05	2.22^*^	0.10	0.05	2.02^*^
Daily work engagement										0.14	0.08	1.71	0.14	0.08	1.74
Daily work engagement*Day-level of WFF													−0.36	0.14	−2.47^**^
−2 X Log(lh)	1,172.34			1,117.20			1,110.55			1,107.62			1,101.58		
Difference of −2 X Log				55.14^***^			6.65^**^			2.93			6.04^*^		
Df				2.00			1.00			1.00			1.00		
Level 1 intercept variance (SE)	0.35 (0.02)			0.35 (0.02)			0.34 (0.02)			0.34 (0.02)			0.34 (0.02)		
Level 2 intercept variance (SE)	0.67 (0.11)			0.38 (0.02)			0.38 (0.06)			0.38 (0.06)			0.37 (0.06)		

*WFF, Work-Family Facilitation*.

* *p <0.05*,

** *p <0.01*,

*** *p <0.001*.

### Detachment From Work

For day-level detachment during off-job time in the evening as an outcome variable (see Table 2), Model 2 provided the best model fit with the positive role of day-level WFF (*B* = 0.13, *SE* = 0.05; *t* = 2.61, *p* < 0.01) contributing to an increased model fit (difference of −2 × log = 6.65, *df* = 1, *p* < 0.01), in line with Hypothesis 1a. However, contrary to Hypothesis 2a, daily work engagement did not have a significant positive relation with detachment from work at night (*B* = 0.14, *SE* = 0.08; *t* = 1.71, *ns*), but Model 3 was not found to be significant (difference of −2 × log = 2.93, *df* = 1, *ns*). Model 4 fit the data significantly better than Model 3 (difference of −2 × log = 6.04, *df* = 1, *p* < 0.05) with the positive relation for day-level WFF (*B* = 0.10, *SE* = 0.05; *t* = 2.05, *p* < 0.05) and the interaction effect on detachment from work during off-job time in the evening. The interaction term between day-level WFF and daily work engagement was significant (*B* = −0.36, *SE* = 0.14; *t* = −2.47, *p* < 0.01).

**Figure 2:**
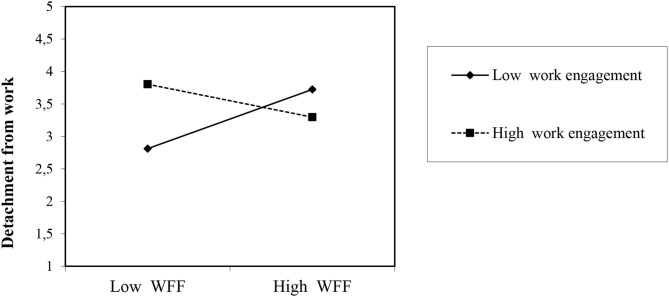
Interaction effects of daily work engagement and day-level of WFF in predicting psychological detachment at night. WFF, Work-Family Facilitation.

To obtain more insight into the role of this interaction, we performed simple slope tests (Preacher et al., 2006). As Figure 2 illustrates, congruent with Hypothesis 3a, the relationship between day-level WFF and detachment from work during off-job time in the evening was stronger when the employee's level of daily work engagement was lower (*y* = −0.629; *SE* = 0.301; *z* = −2.089; *p* < 0.05) rather than higher. Thus, on days on which employees experienced low levels of daily work engagement, day-level WFF was strongly related to detachment from work during off-job time in the evening as opposed to on those days with high levels of daily work engagement. Moreover, surpassing our expectations, simple slope tests showed a significant result for a high slope of daily work engagement (*y* = −0.629; *SE* = 0.301; *z* = −2.089; *p* < 0.05). This result means that on days on which employees experience high levels of daily work engagement, the daily level of WFF was weakly related to detachment from work during off-job time in the evening as opposed to on those days with low levels of daily work engagement at work. With regard to pseudo-*R*^2^, all predictor and control variables entered in the models explained 61.9% of the variance at Level 1 [0.346 – (0.334/0.346) = 0.619] and 14.4% of the variance at Level 2 [0.686 – (0.372/0.686) = 0.144].

### Relaxation

For daily relaxation during off-job time in the evening as an outcome variable (see Table 3), congruent with Hypothesis 1b, Model 2 showed the best model fit with the positive relation for daily WFF (*B* = 0.17, *SE* = 0.05; *t* = 3.33, *p* < 0.001) contributing to an increased model fit (difference of −2 × log = 11.00, *df* = 1, *p* < 0.05). In Model 3, daily work engagement did not have a significant positive relation with relaxation during off-job time in the evening (*B* = 0.14, *SE* = 0.08; *t* = 1.64, *ns*), contrary to Hypothesis 2b, but the model was not found to be significant (difference of −2 × log = 2.70, *df* = 1, *ns*). Model 4 showed a further improvement over Model 3, with daily WFF (*B* = 0.14, *SE* = 0.05; *t* = 2.71, *p* < 0.0*5)* and the interaction term (*B* = −0.45, *SE* = 0.15; *t* = −3.05, *p* < 0.01) was significant.

**Table 3:** Multilevel estimates for models predicting day-level of relaxation (*N* = 520 observations, *N* = 104 participants).

**Variables**	**Null Model**			**Model 1**			**Model 2**			**Model 3**			**Model 4**		
	**Estimate**	**SE**	***t***	**Estimate**	**SE**	***t***	**Estimate**	**SE**	***t***	**Estimate**	**SE**	***T***	**Estimate**	**SE**	***t***
Intercept	3.42	0.08	40.74^***^	3.42	0.07	48.89^***^	3.42	0.07	48.89^***^	3.42	0.07	48.89^***^	3.44	0.07	49.81^***^
General relaxation				0.55	0.08	6.81^***^	0.55	0.08	6.81^***^	0.55	0.08	6.81^***^	0.56	0.08	7.05^***^
Day				0.00	0.02	−0.21	0.00	0.02	0.05	0.00	0.02	0.16	0.01	0.02	0.26
Day-level of WFF							0.17	0.05	3.33^***^	0.15	0.05	2.94^**^	0.14	0.05	2.71^**^
Daily work engagement										0.14	0.08	1.64	0.14	0.08	1.67
Daily work engagement*Day-level of WFF													−0.45	0.15	−3.05^**^
−2 X Log(lh)	1,196.53			1,157.97			1,146.97			1,144.27			1,135.11		
Difference of −2 X Log				38.56^***^			11.00^***^			2.70			9.16^***^		
Df				2.00			1.00			1.00			1.00		
Level 1 intercept variance (SE)	0.37 (0.03)			0.37 (0.03)			0.36 (0.03)			0.36 (0.03)			0.36 (0.02)		
Level 2 intercept variance (SE)	0.67 (0.10)			0.44 (0.07)			0.44 (0.07)			0.44 (0.07)			0.42 (0.07)		

*WFF, Work-Family Facilitation*.

** *p < 0.01*,

*** *p < 0.001*.

**Figure 3:**
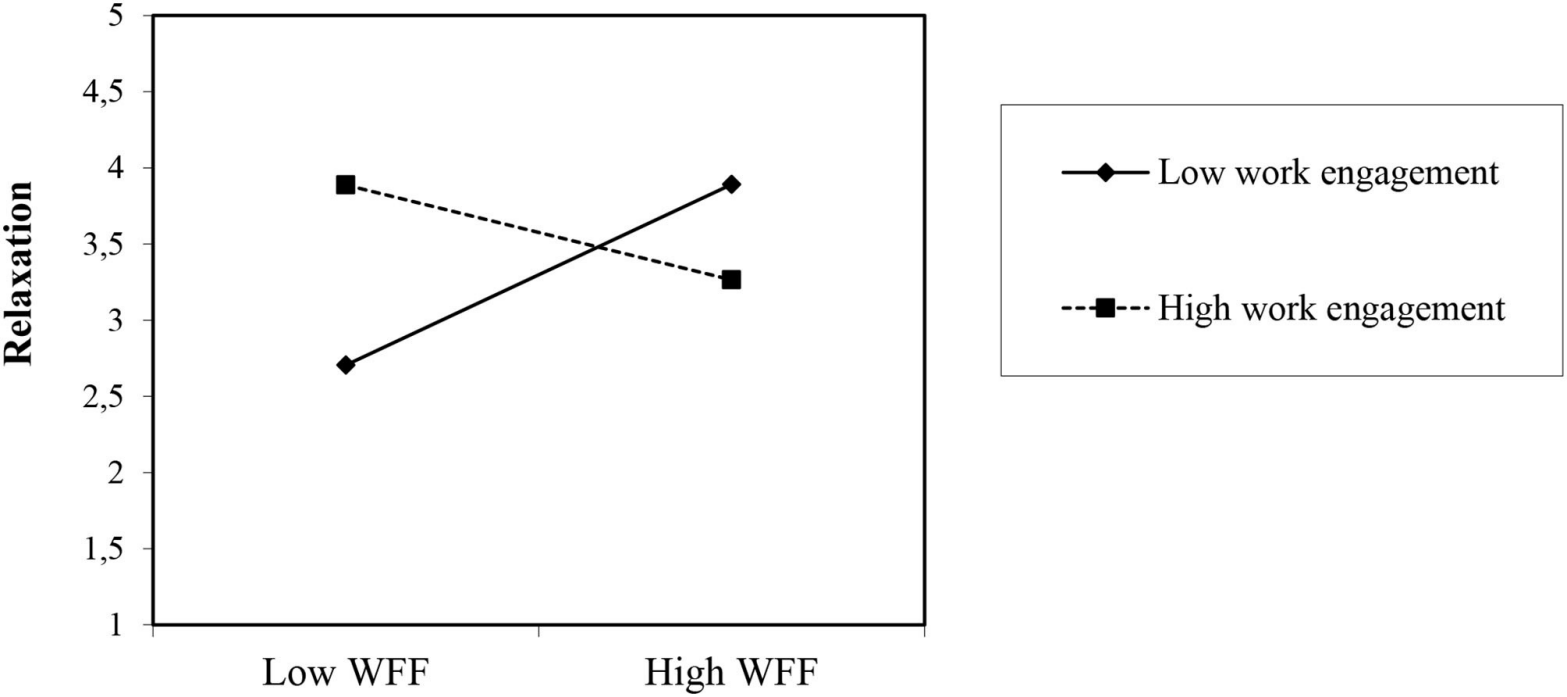
Interaction effects of daily work engagement and day-level of WFF in predicting relaxation at night. WFF, Work-Family Facilitation.

Simple slope tests for the interaction (Preacher et al., 2006) show that congruent with Hypothesis 3b (see Figure 3), the relationship between daily WFF and relaxation during off-job time in the evening was stronger when the employee's daily work engagement was lower (*y* = −0.789; *SE* =0.308; *z* = −2.56; *p* < 0.01) rather than higher. Thus, on days on which employees experienced low levels of daily work engagement, the daily WFF was strongly related to relaxation during off-job time in the evening as opposed to on those days with high levels of daily work engagement. Supporting our expectations, simple slope tests show a significant result for a high level of daily work engagement (*y* = −2.092; *SE* = 0.724; *z* = −2.88; *p* < 0.01). This result shows that on days on which employees experienced high levels of daily work engagement, daily WFF was weakly related to relaxation during off-job time in the evening as opposed to on those days with low levels of daily work engagement. With regard to pseudo-*R*^2^, all predictor and control variables entered in the models explained 58.7% of the variance at Level 1 [0.369 – (0.353/0.369) = 0.587] and 4% of the variance at Level 2 [0.665 – (0.416/0.665) = 0.040].

### General Health

**Figure 4:**
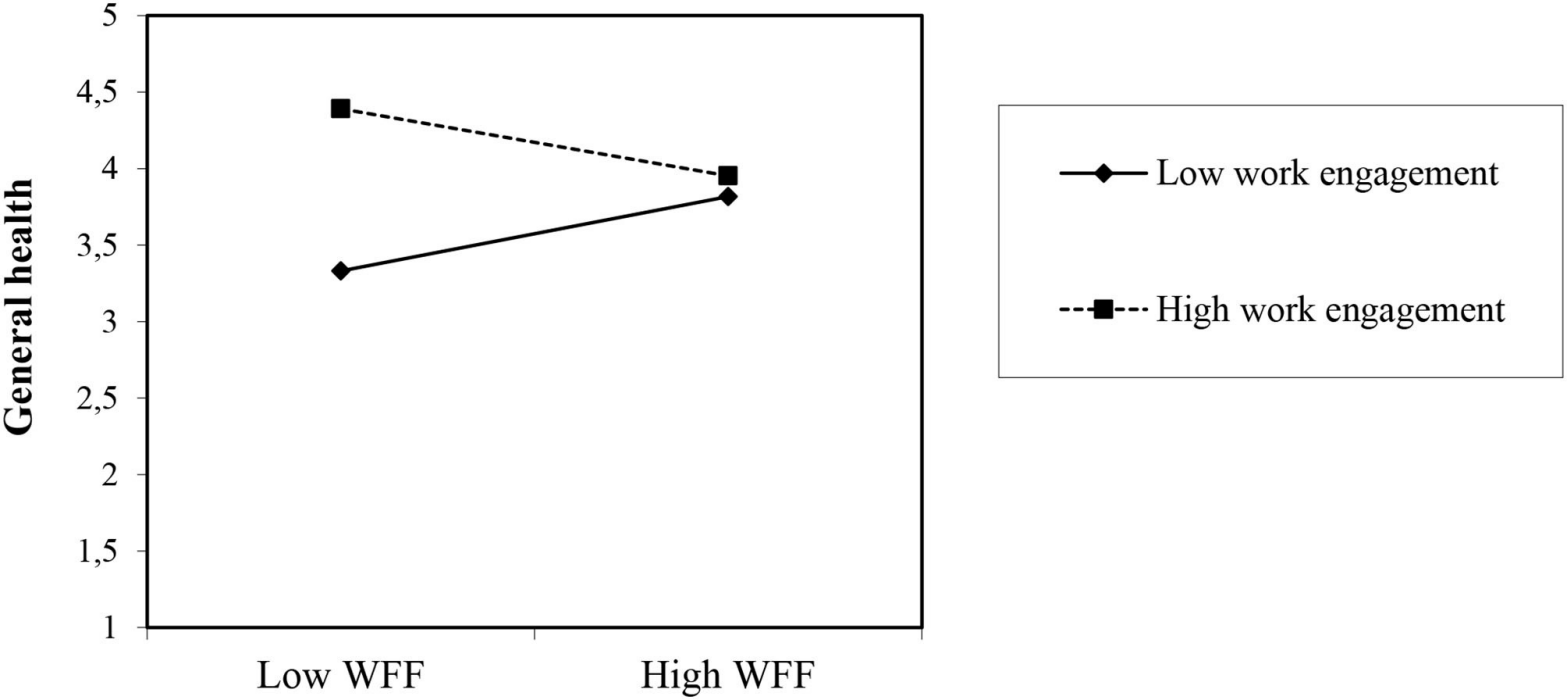
Interaction effects of daily work engagement and day-level of WFF in predicting general health at night. WFF, Work-Family Facilitation.

For daily general health at night (see Table 4), Model 2 was not found to be significant (difference of −2 × log = 3.34, *df* = 1, *ns*), and Hypothesis 1c is not supported. Daily WFF did not have a significant positive relation with general health at night (*B* = 0.06, *SE* = 0.03; *t* = 1.83, *ns*). However, Model 3 provided the best model fit with the positive relation for daily work engagement (*B* = 0.30, *SE* = 0.05; *t* = 6.36, *p* < 0.001) contributing to an increased model fit (difference of −2 × log = 37.91, *df* = 1, *p* < 0.001), in line with Hypothesis 2c. Model 4 fit the data significantly (difference of −2 × log = 7.61, *df* = 1, *p* <0.01) with the positive relation for daily work engagement (*B* = 0.30, *SE* = 0.05; *t* = 6.36, *p* < 0.001) and the interaction effect between daily WFF and work engagement on general health at night (see Figure 4) (*B* = −0.46, *SE* = 0.08; *t* = −5.57, *p* < 0.001).

**Table 4:** Multilevel estimates for models predicting day-level of general health (*N* = 520 observations, *N* = 104 participants).

**Variables**	**Null Model**			**Model 1**			**Model 2**			**Model 3**			**Model 4**		
	**Estimate**	**SE**	***t***	**Estimate**	**SE**	***t***	**Estimate**	**SE**	***t***	**Estimate**	**SE**	***t***	**Estimate**	**SE**	***t***
Intercept	3.87	0.06	67.81^***^	3.87	0.04	104.46^***^	3.87	0.04	104.46^***^	3.87	0.04	104.46^***^	3.87	0.04	104.68^***^
Trait of general health				0.75	0.06	11.89^***^	0.75	0.06	11.89^***^	0.75	0.06	11.89^***^	0.75	0.06	11.83^***^
Day				−0.02	0.01	−1.91	−0.02	0.01	−1.82	−0.01	0.01	−1.27	−0.01	0.01	−1.30
Day-level of WFF							0.06	0.03	1.83	0.02	0.03	0.62	0.01	0.03	0.41
Daily work engagement										0.30	0.05	6.36^***^	0.30	0.05	6.36^***^
Daily work engagement*Day-level of WFF													−0.46	0.08	−5.57^***^
−2 X Log(lh)	671.01			577.06			573.72			535.81			528.20		
Difference of −2 X Log				93.95^***^			3.34			37.91^***^			7.61^**^		
Df				2.00			1.00			1.00			1.00		
Level 1 intercept variance (SE)	0.13 (0.01)			0.13 (0.01)			0.13 (0.01)			0.12 (0.01)			0.11 (0.01)		
Level 2 intercept variance (SE)	0.31 (0.05)			0.12 (0.02)			0.18 (0.02)			0.18 (0.02)			0.12 (0.02)		

*WFF, Work-Family Facilitation*.

** *p < 0.01*,

*** *p < 0.001*.

Following Preacher et al. (2006) and congruent with Hypothesis 3c (see Figure 4), simple slope tests for the interaction show that the relationship between daily WFF and general health at night was stronger when the employee's daily work engagement was lower (*y* = −0.939; *SE* =0.175; *z* = −5.36; *p* < 0.001) rather than higher. Thus, on days on which employees experienced low levels of daily work engagement, daily WFF was strongly related to general health at night as opposed to on those days with high levels of daily work engagement. Additionally, simple slope tests show a significant result for a high level of daily work engagement at work (*y* = −2.270; *SE* = 0.414; *z* = −5.47; *p* < 0.001), which exceeds our expectations. This means that on days on which employees experienced high levels of daily work engagement, daily WFF was weakly related to general health at night as opposed to on those days with low levels of daily work engagement. With regard to pseudo-*R*^2^, all predictor and control variables entered in the models explained 74.7% of the variance at Level 1 [0.127 – (0.111/0.127) = 0.747] and 7.5% of the variance at Level 2 [0.312 – (0.121/0.312) = 0.075].

### Next-Day General Health

For predicting day-level general health at night on the following day (see Table 5), according to Hypothesis 4, day-level detachment from work during the previous evening during off-job time (day 1) showed a positive relationship to next-day general health at night (*B* = 0.07, *SE* = 0.03; *t* = 2.18, *p* < 0.05), and Model 2 also provided a significant model fit (difference of −2 × log = 4.72, *df* = 1, *p* < 0.05). With regard to pseudo-*R*^2^, all predictor and control variables entered in the models explained 84.6% of the variance at Level 1 [0.130 – (0.127/0.130) = 0.846] and 6.1% of the variance at Level 2 [0.311 – (0.116/0.311) = 0.061].

**Table 5:** Multilevel estimates for models predicting day-level of next-day general health (*N* = 520 observations, *N* = 104 participants).

**Variables**	**Null Model**			**Model 1**			**Model 2**		
	**Estimate**	**SE**	***t***	**Estimate**	**SE**	***t***	**Estimate**	**SE**	***t***
Intercept	3.88	0.06	68.00^***^	3.89	0.04	102.29^***^	3.89	0.04	102.26^***^
Trait of general health				0.75	0.06	11.73^***^	0.75	0.06	11.72^***^
Day				−0.02	0.02	−1.38	−0.02	0.02	−1.38
Day-level of detachment from work in day 1							0.07	0.03	2.18^*^
−2 X Log(lh)	577.52			487.50			482.78		
Difference of −2 X Log				90.02^***^			4.72^*^		
Df				2.00			1.00		
Level 1 intercept variance (SE)	0.13 (0.01)			0.13 (0.01)			0.13 (0.01)		
Level 2 intercept variance (SE)	0.31 (0.05)			0.12 (0.02)			0.12 (0.02)		

* *p < 0.05*,

*** *p < 0.001*.

## Discussion

The present study investigates the process of daily WFF—recovery—general health, which to our knowledge has not been considered before. The results mostly confirm our hypotheses. As expected, daily WFF predicted detachment from work and relaxation (as recovery experiences) during off-job time in the evening, while daily work engagement only predicted general health at night, contrary to our expectations. Moreover, as expected, a moderation effect of daily work engagement showed that on days on which employees experienced low levels of daily work engagement, daily WFF was strongly related to detachment from work and relaxation during off-job time in the evening and to general health at night. Unexpectedly, on days on which employees experienced high levels of daily work engagement, daily WFF was weakly related to these outcomes. Finally, in accordance with our expectations, detachment from work had a lagged effect on next-day general health at night. These findings show that daily WFF can trigger recovery experiences in the evening, and daily work engagement can foster general health at night. However, only a low level of daily work engagement is necessary to recover and experience general health for employees high in WFF. Moreover, the benefits of detachment from work can extend to the following day at night. The diary design of our study leads us to an intrapersonal explanation of the process of WFF—recovery—general health and accounts for both the work and home spheres in studying positive spillover in natural scenarios among Chilean employees.

### Theoretical Implications

We used the W-HR model (ten Brummelhuis and Bakker, 2012) to explain the daily process of WFF—recovery—general health among Chilean employees. The aim of the present study was threefold. Our first goal was to test whether the beneficial effect of daily WFF and daily work engagement impacts recovery experiences during off-job time in the evening and subsequently employees' general health at night. Multilevel analyses show positive direct effects of daily WFF on detachment from work and relaxation but not on employees' general health at night. Our results extend previous research on the positive synergistic potential of work to home, which highlights the positive view of daily WFF as an antecedent of recovery experiences. Most research focuses on the positive side of WFF in terms of well-being outcomes (Wendsche and Lohmann-Haislah, 2017; Bennett et al., 2018; Carlson et al., 2019; Jiao and Lee, 2020), while less attention has been given to exploring the positive aspects of WFF as an antecedent of recovery. Our results reveal that WFF is crucial to the day-to-day recovery process, implying that employees who achieve a positive transference of system functioning from work to the home domain may experience a daily process of restoring resources during off-job time. According to the W-HR model (ten Brummelhuis and Bakker, 2012), daily WFF as part of a process of resource accumulation contributes to individual psychological well-being. Thus, our findings support this model by means of the intrapersonal process of recovery, which is influenced by the positive short-term gain spirals of daily WFF.

Unexpectedly, our findings do not support our postulates related to the W-HR model, and we found that daily WFF does not have a significant positive relationship with employees' general health at night, even though it is considered a precursor of health and well-being (Jones et al., 2020), while it is significant in predicting recovery. In contrast, daily work engagement is a precursor of employees' general health at the end of the day but does not predict recovery. These results run contrary to previous evidence showing a reciprocal relation between daily work engagement and recovery (Bakker, 2014). Surprisingly, these findings could indicate the presence of different daily mechanisms depending on the nature of psychological constructs involved. It seems that the positive interaction between work and the family sphere by means of the work sphere facilitates system functioning at home and improves aspects in terms of performance. That is, people can feel better by engaging in activities with a partner, family or friends; achieving better performance in domestic tasks; or duly fulfilling family responsibilities because they have acquired the required skills or competencies in the work sphere, allowing them to recover well. In contrast, the beneficial effects of daily WFF might not be related to general health because they could depend on other diary aspects. Examples may include other contextual variables of work that have not been measured or personal variables such as emotional regulation and personality characteristics. Thus, in line with the W-HR model developed by ten Brummelhuis and Bakker (2012), contextual work resources seem to be relevant to gaining short-term personal resource processes.

Our second goal was to examine the moderating role of daily work engagement in the relationship between recovery experiences and employees' general health. The results indicate that on days on which employees are low in daily work engagement, the daily level of WFF is strongly related to recovery and general health. Our results also exceed expectations by showing that on days on which employees are high in daily work engagement, the daily level of WFF is weakly related to recovery and general health. Curiously, these results seem to be in line with the debate on a “dark side of engagement,” which posits that employees could ascribe excessive importance to their work that impacts their lives (Bakker and Leiter, 2010; George, 2010; Bakker et al., 2011; Leiter, 2019; Blanco-Donoso et al., 2021). In the case of our study, excessive daily work engagement might be an obstacle to recovering and experiencing general health at the end of the day. What our results seem to indicate is that when employees have already earned positive transference from work competencies to implementation in home-related tasks, it might not be necessary to be highly engaged during the workday. Thus, in accordance with emerging evidence, daily work engagement may have curvilinear—instead of linear—effects on recovery and mental health (Shimazu et al., 2018).

Finally, our third goal was to shed light on the lagged effect of psychological detachment from work as a core recovery experience. Multilevel results show that its positive impact on employees persists to next-day general health at night. Employees who detached from work during off-job time the previous evening experienced better general health at bedtime on the following day. Most research on psychological detachment from work has focused on its mediational role (Sonnentag et al., 2010a; Sonnentag and Fritz, 2015; Sonnentag and Lischetzke, 2018). Despite the relevance of this knowledge for theory and practice, our study suggests the relevance of exploring lagged effects that might have an association with an individual's well-being. Until now, few studies have explored the lagged effect of detachment from work at the daily level, and our results not only are congruent with previous evidence on its benefits the following morning (Sonnentag et al., 2008; ten Brummelhuis and Bakker, 2012; Volman et al., 2013; Pereira et al., 2016) but also go further by measuring the effect on the following day at bedtime.

### Implications for Practice

The importance of the beneficial effects of day-level WFF on daily recovery experiences is supported by this study. Theoretically, this study is based on the W-HR model focusing on the positive synergistic potential of the work–family interface. Due to the benefits of daily WFF for recovery experiences, employees should be informed about the potential positive effect of daily processes on their lives to learn how to regulate their work and home spheres and to become aware of the importance of actively seeking recovery experiences. Following experts' recommendations, inclusive organizations should provide employees with resources to effectively perform in the private sphere, fostering daily recovery from work stress (Martinez-Corts and Demerouti, 2017). Thus, we encourage organizations to evaluate psychosocial risks using specific measures [for example, see Wood et al. (2019)] and to even expand training and coaching programs, for example, by integrating a preventive module on healthy work-home balance techniques. In such training [for example, see Bisschoff et al. (2019) and Wu and Chang (2020)], employees should be asked to apply gains acquired at work to the home sphere to be more efficient during off-job time and recover well. Employees can offset the gains of the work domain (e.g., management strategies to very high job demands) by using opportunities for recovery during daily off-job time.

According to our findings, in such training, employees should also be urged to implement strategies promoting psychological detachment from work during their off-job time. For example, a recent intervention revealed that employees with more leader-member exchange and a higher need for recovery benefit most from learning strategies for detaching at work (Clauss et al., 2018). Furthermore, organizations can adopt mindfulness training at the team and individual levels to regulate employees' levels of work engagement because its positive influence may be helpful in affecting the curvilinear trend of work engagement (Liu et al., 2020). Doing so in an early stage might have beneficial effects on employees' general health and in managing healthy organizations. These practical implications are particularly beneficial for employees who have to deal with emerging trends in current job-related demands, such as telepressure and technostress, which make work–life balance and employee well-being more difficult to achieve (Barber et al., 2019; Pfaffinger et al., 2020).

### Limitations and Future Research

This research has several limitations. The first limitation stems from the self-report measures used. These measures relied on the sincerity of the participants' responses and may have been subject to social desirability bias. Although researchers provided each of the participants with an envelope to maintain their privacy and confidentiality, in future studies, it might help to incorporate the use of online questionnaires instead of paper surveys and envelopes to improve the anonymity of data collection. Another limitation relates to the variety of occupations our participants were engaged in (e.g., services, administration, banking, Information Technology and Communications, and education). The participants were recruited *via* snowball sampling, but other sampling techniques could be used to render sociodemographic characteristics uniform in future research. In addition, while women and men differ in their experiences of combined conflict and facilitation balance (Boz et al., 2016), this study did not adopt a gendered perspective. Future studies should differentially analyze the daily process of WFF—recovery—general health for female and male Chilean employees to better understand the specific relationships involved, as well as considering socio-demographic variables such the age of employees' children. Moreover, considering the relevance of specific recovery experiences, future studies should also focus on other types of recovery experiences, such as mastery. The present study involved collecting data before the COVID-19 outbreak. Future research could further pursue daily process exploration to understand how pandemic measures affect working modes (e.g., smart working and the closure of schools with subsequent effects for working parents), which may have an impact on the beneficial effects of daily WFF.

## Conclusion

As this diary study shows, recovery experiences depend on how employees manage their daily work to home balance. On days on which employees are high in WFF, they report stronger recovery experiences. Low daily work engagement moderates the relationship between daily WFF and recovery and subsequent general health, while high daily work engagement is an obstacle within these relationships. Moreover, in line with previous research, we conclude that psychological detachment from work is the core recovery experience that persists to next-day general health at night. In sum, we encourage researchers to further explore the daily process of enrichment—recovery—general health to build on healthy strategies that allow employees to achieve work-life balance and recover from their daily job demands.

## Data Availability Statement

The raw data supporting the conclusions of this article will be made available by the authors, without undue reservation.

## Ethics Statement

The studies involving human participants were reviewed and approved by Ethics committee of the Catholic University of Temuco. The patients/participants provided their written informed consent to participate in this study.

## Author Contributions

IC-C focused on the design and data collection, the analysis and interpretation of the results, and the drafting of the paper. LB-D participated in the analysis and interpretation of the results, as well as in different revisions of the paper during its realization. EG participated in the design of the study and in different revisions of the paper during its realization. All authors of this original paper have directly participated in the planning, execution, or analysis of this research, as well as read and approved the final version submitted.

## Conflict of Interest

The authors declare that the research was conducted in the absence of any commercial or financial relationships that could be construed as a potential conflict of interest.

## References

[B1] Araya R. Wynn R. Lewis G. (1992). Comparison of two self administered psychiatric questionnaires (GHQ-12 and SRQ-20) in primary care in Chile. Soc. Psychiatry Psychiatr. Epidemiol. 27, 168–173. 10.1007/BF00789001 1411744

[B2] Bakker A. B. (2014). Daily fluctuations in work engagement: an overview and current directions. Eur. Psychol. 19, 227–236. 10.1027/1016-9040/a000160

[B3] Bakker A. B. Albrecht S. (2018). Work engagement: current trends. Career Dev. Int. 23, 4–11. 10.1108/CDI-11-2017-0207

[B4] Bakker A. B. Albrecht S. L. Leiter M. P. (2011). Work engagement: further reflections on the state of play. Eur. J. Work Organ. Psychol. 20:7488. 10.1080/1359432X.2010.546711

[B5] Bakker A. B. Leiter M. P. (2010). Where to go from here: integration and future research on work engagement, in Work Engagement: A Handbook of Essential Theory and Research, eds Bakker A. B. Leiter M. P. (New York, NY: Psychology Press), 181–96. 10.4324/9780203853047

[B6] Barber L. K. Conlin A. L. Santuzzi A. M. (2019). Workplace telepressure and work–life balance outcomes: the role of work recovery experiences. Stress Health 35, 350–362. 10.1002/smi.2864 30882979

[B7] Bennett A. A. Bakker A. B. Field J. G. (2018). Recovery from work-related effort: a meta-analysis. J. Organ. Behav. 39, 262–275. 10.1002/job.2217

[B8] Bisschoff M. Koen V. Ryke E. H. (2019). Strategies for work–family balance in a South African context. Commun. Work Family 22, 319–337. 10.1080/13668803.2018.1473337

[B9] Blanco-Donoso L. M. Garrosa E. Demerouti E. Moreno-Jiménez B. (2017). Job resources and recovery experiences to face difficulties in emotion regulation at work: a diary study among nurses. Int. J. Stress Manage. 24, 107–134. 10.1037/str0000023

[B10] Blanco-Donoso L. M. Moreno-Jiménez J. Amutio A. dos Santos M. Garrosa E. (2021). Overwhelmed by emotional job demands in high vigor days! its detrimental effects on daily recovery from work among health-care workers. J. Psychol. 2020:1870910. 10.1080/00223980.2020.1870910 33539273

[B11] Bolger N. Davis A. Rafaeli E. (2003). Diary methods: capturing life as it is lived. Annu. Rev. Psychol. 54, 579–616. 10.1146/annurev.psych.54.101601.145030 12499517

[B12] Boz M. Martínez-Corts I. Munduate L. (2016). Types of combined family-to-work conflict and enrichment and subjective health in Spain: a gender perspective. Sex Roles 74, 136–153. 10.1007/s11199-015-0461-5

[B13] Carlson D. S. Thompson M. J. Crawford W. S. Kacmar K. M. (2019). Spillover and crossover of work resources: a test of the positive flow of resources through work–family enrichment. J. Organ. Behav. 40, 709–722. 10.1002/job.2363

[B14] Chawla N. MacGowan R. L. Gabriel A. S. Podsakoff N. P. (2020). Unplugging or staying connected? Examining the nature, antecedents, and consequences of profiles of daily recovery experiences. J. Appl. Psychol. 105, 19–39. 10.1037/apl0000423 31204831

[B15] Clauss E. Hoppe A. Schachler V. (2018). Promoting psychological detachment and detachment-related self-efficacy through an online training (WITHDRAWN). Proc. Acad. Manag. N. Y. 1:12021. 10.5465/AMBPP.2018.12021abstract

[B16] Clauss E. Hoppe A. Schachler V. O'Shea D. (2020). Occupational self-efficacy and work engagement as moderators in the stressor-detachment model. Work Stress 2020, 1–19. 10.1080/02678373.2020.1743790

[B17] Fisher C. D. To M. L. (2012). Using experience sampling methodology in organizational behavior. J. Organ. Behav. Manage. 33, 865–877. 10.1002/job.1803

[B18] Frone M. R. (2003). Work-family balance, in Handbook of Occupational Health Psychology, eds Quick J. C. Tetrick L. E. (Washington, D.C: American Psychological Association), 143–162. 10.1037/10474-007

[B19] Garrosa E. Blanco-Donoso L. M. Carmona-Cobo I. Moreno-Jiménez B. (2017). How do curiosity, meaning in life, and search for meaning predict college students' daily emotional exhaustion and engagement? J. Happiness Stud. 18, 17–40. 10.1007/s10902-016-9715-3

[B20] Garrosa E. Carmona-Cobo I. Moreno-Jiménez B. Sanz-Vergel A. I. (2015). El impacto emocional del incivismo laboral y el abuso verbal en el trabajo: influencia de la recuperación diaria. An. Psicol. 31, 190–198. 10.6018/analesps.31.1.161491

[B21] Garrosa-Hernández E. Carmona-Cobo I. Ladstätter F. Blanco L. M. Cooper-Thomas H. D. (2013). The relationships between family-work interaction, job-related exhaustion, detachment, and meaning in life: a day-level study of emotional well-being. Revista de Psicología del Trabajo y de las Organizaciones 29, 169–177. 10.5093/tr2013a23

[B22] George J. (2010). More engagement is not necessarily better: the benefits of fluctuating levels of engagement, in Handbook of Employee Engagement: Perspectives, Issues, Research and Practice, ed Albrecht S. L. (Glos, UK: Edwald Elgar), 253–263.

[B23] Geurts S. A. Taris T. W. Kompier M. A. Dikkers J. S. Van Hooff M. L. Kinnunen U. M. (2005). Work-home interaction from a work psychological perspective: development and validation of a new questionnaire, the SWING. Work Stress 19, 319–339. 10.1080/02678370500410208

[B24] Greenhaus J. H. Powell G. N. (2006). When work and family are allies: A theory of work-family enrichment. Acad. Manage. Rev. 31, 72–92. 10.5465/amr.2006.19379625

[B25] Grzywacz J. G. Marks N. F. (2000). Reconceptualizing the work–family interface: an ecological perspective on the correlates of positive and negative spillover between work and family. J. Occup. Health Psychol. 5, 111–126. 10.1037/1076-8998.5.1.111 10658890

[B26] Hambleton R. K. De Jong J. H. A. L. (2003). Advances in translating and adapting educational and psychological tests. Lang. Test. 20, 127–134. 10.1191/0265532203lt247xx

[B27] Hobfoll S. E. (2001). The influence of culture, community, and the nested-self in the stress process: advancing conservation of resources theory. Appl. Psychol. 50, 337–421. 10.1111/1464-0597.00062

[B28] Hox J. Roberts J. K. (2011). Multilevel analysis: where we were and where we are, in Handbook of Advanced Multilevel Analysis, eds Hox J. J. Roberts J. K. (New York, NY: Routledge), 3–11. 10.4324/9780203848852

[B29] Hox J. J. (2010). Multilevel Analysis: Techniques and Applications. New York, NY: Routledge.

[B30] Jeffrey Hill E. Yang C. Hawkins A. J. Ferris M. (2004). A cross-cultural test of the work-family interface in 48 countries. J Marriage Fam. 66, 1300–1316. 10.1111/j.0022-2445.2004.00094.x

[B31] Jiao P. Lee C. (2020). Perceiving a resourcefulness: longitudinal study of the sequential mediation model linking between spiritual leadership, psychological capital, job resources, and work-to-family facilitation. Front. Psychol. 11:613360. 10.3389/fpsyg.2020.613360 33643115 PMC7906966

[B32] Jiménez Figueroa A. Gómez Urrutia V. (2014). Family responsibility, organizational practices, work-family balance and subjective welfare in Chile. Civilizar 14, 85–96. 10.22518/16578953.181

[B33] Jiménez Figueroa A. Gómez Urrutia V. Palomo-Vélez G. (2017). Work-family balance, participation in family work and parental self-efficacy in Chilean workers. Can. J. Sch. Psychol. 9, 1–25. 10.29173/cjfy29007

[B34] Jones B. L. Hill E. J. Miller R. B. (2020). Family routines and family satisfaction in Singapore: work–family fit as a moderator. Asia Pac. J. Hum. Resour. 58, 24–45. 10.1111/1744-7941.12215

[B35] Leckie G. Charlton C. (2013). Runmlwin-a program to Run the MLwiN multilevel modelling software from within stata. J. Stat. Softw. 52, 1–40. 10.18637/jss.v052.i1123761062

[B36] Leiter M. (2019). The psychology of work engagement. Oxford Res. Encyclop. Psychol. 13:36. 10.1093/acrefore/9780190236557.013.36

[B37] Liu S. Xin H. Shen L. He J. Liu J. (2020). The influence of individual and team mindfulness on work engagement. Front. Psychol. 10:2928. 10.3389/fpsyg.2019.02928 32038356 PMC6985205

[B38] Martinez-Corts I. Demerouti E. (2017). Developing Multiple Careers: Dealing with Work–Life Interaction, in Shaping Inclusive Workplaces Through Social Dialogue, eds Arenas A. Di Marco D. Munduate L. Euwema M. . (Springer, Cham), 221–237. 10.1007/978-3-319-66393-7_15

[B39] Moreno-Jiménez B. Sanz-Vergel A. I. Rodríguez-Muñoz A. Geurts S. (2009). Propiedades psicométricas de la versión española del Cuestionario de Interacción Trabajo-Familia (SWING). Psicothema 21, 331–337. 19403091

[B40] Müller Gilchrist R. Pérez Villalobos C. E. Ramírez Fernández L. (2013). Estructura factorial y consistencia interna de la Utrech Work Engagement Scale (UWES) 17 entre trabajadores sanitarios de Chile. Liberabit 19, 163–171.

[B41] Nezlek J. B. (2012). The Sage Library of Methods in Social and Personality Psychology: Diary Methods for Social and Personality Psychology. SAGE Publications. 10.4135/9781446287903

[B42] Ohly S. Sonnentag S. Niessen C. Zapf D. (2010). Diary studies in organizational research. J. Pers. Psychol 9, 79–93. 10.1027/1866-5888/a000009

[B43] Orellana L. Schnettler B. Miranda-Zapata E. Lobos G. Lapo M. Adasme-Berríos C.. (2021). Resource transmission is not reciprocal: a dyadic analysis of family support, work-life balance, and life satisfaction in dual-earner parents with adolescent children. Sex Roles 20, 1–12. 10.1007/s11199-020-01207-0

[B44] Ouweneel E. Le Blanc P. M. Schaufeli W. B. van Wijhe C. I. (2012). Good morning, good day: a diary study on positive emotions, hope, and work engagement. Hum. Relat. 65, 1129–1154. 10.1177/0018726711429382

[B45] Parker S. L. Sonnentag S. Jimmieson N. L. Newton C. J. (2020). Relaxation during the evening and next-morning energy: the role of hassles, uplifts, and heart rate variability during work. J. Occup. Health Psychol. 25, 83–98. 10.1037/ocp0000155 31219269

[B46] Pereira D. Bucher S. Elfering A. (2016). Daily impaired detachment and short-term effects of impaired sleep quality on next-day commuting near-accidents–an ambulatory diary study. Ergonomics 59, 1121–1131. 10.1080/00140139.2015.1115898 27049337

[B47] Pfaffinger K. F. Reif J. A. Spieß E. (2020). When and why telepressure and technostress creators impair employee well-being. Int. J. Occup. Saf. Ergon. 2020, 1–16. 10.1080/10803548.2020.1846376 33164707

[B48] Preacher K. J. Curran P. J. Bauer D. J. (2006). Computational tools for probing interactions in multiple linear regression, multilevel modeling, and latent curve analysis. J. Educ. Behav. Stat. 31, 437–448. 10.3102/10769986031004437

[B49] Rodríguez-Muñoz A. Sanz-Vergel A. I. Antino M. Demerouti E. Bakker A. B. (2018). Positive experiences at work and daily recovery: effects on couple's well-being. J. Happiness Stud. 19, 1395–1413. 10.1007/s10902-017-9880-z

[B50] Rodríguez-Muñoz A. Sanz-Vergel A. I. Demerouti E. Bakker A. B. (2012). Reciprocal relationships between job demands, job resources, and recovery opportunities. J. Pers. Psychol. 11, 86–94. 10.1027/1866-5888/a000049

[B51] Sanz-Vergel A. I. Demerouti E. Moreno-Jiménez B. Mayo M. (2010a). Work-family balance and energy: a day-level study on recovery conditions. J. Vocat. Behav. 76, 118–130. 10.1016/j.jvb.2009.07.001

[B52] Sanz-Vergel A. I. Sebastián J. Rodríguez-Muñoz A. Garrosa E. Moreno-Jiménez B. Sonnentag S. (2010b). Adaptación del “Cuestionario de Experiencias de Recuperación” a una muestra española. Psicothema 22, 990–996. 21044543

[B53] Schaufeli W. B. Bakker A. B. Salanova M. (2006). The measurement of work engagement with a short questionnaire: a cross-national study. Educ. Psychol. Meas. 66, 701–716. 10.1177/0013164405282471

[B54] Shimazu A. Schaufeli W. B. Kubota K. Watanabe K. Kawakami N. (2018). Is too much work engagement detrimental? Linear or curvilinear effects on mental health and job performance. PLoS ONE. 13:e0208684. 10.1371/journal.pone.0208684 30586369 PMC6306155

[B55] Singer J. D. Willett J. B. (2003). Applied Longitudinal Data Analysis: Modeling Change and Event Occurrence. Oxford: Oxford Univ. Press.

[B56] Sonnentag S. (2003). Recovery, work engagement, and proactive behavior: a new look at the interface between nonwork and work. J. Appl. Psychol. 88, 518–528. 10.1037/0021-9010.88.3.518 12814299

[B57] Sonnentag S. Binnewies C. Mojza E. J. (2008). Did you have a nice evening?” A day-level study on recovery experiences, sleep, and affect. J. Appl. Psychol. 93, 674–684. 10.1037/0021-9010.93.3.674 18457495

[B58] Sonnentag S. Binnewies C. Mojza E. J. (2010a). Staying well and engaged when demands are high: the role of psychological detachment. J. Appl. Psychol. 95, 965–976. 10.1037/a0020032 20718528

[B59] Sonnentag S. Dormann C. Demerouti E. (2010b). Not all days are created equal: the concept of state work engagement, in Work Engagement: A Handbook of Essential Theory and Research, eds Bakker A. B. Leiter M. P. (New York, NY: Psychology Press), 25–38.

[B60] Sonnentag S. Fritz C. (2007). The Recovery Experience Questionnaire: development and validation of a measure for assessing recuperation and unwinding from work. J. Occup. Health Psychol. 12, 204–221. 10.1037/1076-8998.12.3.204 17638488

[B61] Sonnentag S. Fritz C. (2015). Recovery from job stress: the stressor-detachment model as an integrative framework. J. Organ. Behav. 36, S72–S103. 10.1002/job.1924

[B62] Sonnentag S. Lischetzke T. (2018). Illegitimate tasks reach into afterwork hours: a multilevel study. J. Occup. Health Psychol. 23, 248–261. 10.1037/ocp0000077 28206790

[B63] Sonnentag S. Mojza E. J. Demerouti E. Bakker A. B. (2012). Reciprocal relations between recovery and work engagement: the moderating role of job stressors. J. Appl. Psychol. 97, 842–853. 10.1037/a0028292 22545619

[B64] Sonnentag S. Niessen C. (2020). To detach or not to detach? two experimental studies on the affective consequences of detaching from work during non-work time. Front. Psychol. 11:560156. 10.3389/fpsyg.2020.560156 33178068 PMC7596587

[B65] Staab S. (2012). Maternalism, male breadwinner bias, and market reform: historical legacies and current reforms in Chilean social policy. Soc. Politics 19, 299–332. 10.1093/sp/jxs010

[B66] ten Brummelhuis L. L. Bakker A. B. (2012). A resource perspective on the work–home interface: the work–home resources model. Am. Psychol. 67, 545–556. 10.1037/a0027974 22506688

[B67] Vogt D. S. King D. W. King L. A. (2004). Focus groups in psychological assessment: enhancing content validity by consulting members of the target population. Psychol. Assess. 16, 231–243. 10.1037/1040-3590.16.3.231 15456379

[B68] Volman F. E. Bakker A. B. Xanthopoulou D. (2013). Recovery at home and performance at work: a diary study on self–family facilitation. Eur. J. Work Organ. Psychol. 22, 218–234. 10.1080/1359432X.2011.648375

[B69] Wayne J. H. (2009). Reducing conceptual confusion: clarifying the positive side of work and family, in Handbook of Families and Work: Interdisciplinary Perspectives, eds Russell Crane D. Jeffrey E. Hill (University Press of America), 105–140.

[B70] Wayne J. H. Grzywacz J. G. Carlson D. S. Kacmar K. M. (2007). Work–family facilitation: a theoretical explanation and model of primary antecedents and consequences. Hum. Resour. Manag. Rev. 17, 63–76. 10.1016/j.hrmr.2007.01.002

[B71] Wendsche J. Lohmann-Haislah A. (2017). A meta-analysis on antecedents and outcomes of detachment from work. Front. Psychol. 7:2072. 10.3389/fpsyg.2016.02072 28133454 PMC5233687

[B72] Willgerodt M. A. (2003). Using focus groups to develop culturally relevant instruments. West. J. Nurs. Res. 25, 798–814. 10.1177/0193945903256708 14596180

[B73] Wood S. Ghezzi V. Barbaranelli C. Di Tecco C. Fida R. Farnese M. L.. (2019). Assessing the risk of stress in organizations: getting the measure of organizational-level stressors. Front. Psychol. 10:2776. 10.3389/fpsyg.2019.02776 31920825 PMC6932998

[B74] Wu T. Chang P. C. (2020). The impact of work–family programs on work–family facilitation and role performance: the dual moderating effect of gender. Asia Pacific J. Hum. Resour. 58, 46–65. 10.1111/1744-7941.12206

[B75] Zijlstra F. R. Sonnentag S. (2006). After work is done: psychological perspectives on recovery from work. Eur. J. Work Organ. Psychol. 15, 129–138. 10.1080/13594320500513855

